# Supplementary material for: Collaborative Ring Trial of the Applicability of a Reference Plasmid DNA Calibrant in the Quantitative Analysis of GM Maize Event MON810
Source: Foods. 2022 May 24;11(11):1538. doi: 10.3390/foods11111538 (PMC9180190; doi:10.3390/foods11111538)
Supplement: Supplementary file 1 [file foods-11-01538-s001.zip › foods-1690502-supplementary.pdf]

foods

Supplementary Materials

Article

Collaborative ring trial of the applicability of a reference plasmid DNA calibrant in the quantitative analysis of GM maize event MON810

Yanan Meng[1,2], Shu Wang[2], Jinchao Guo[2] and Litao Yang[2*]

[1] Pilot National Laboratory for Marine Science and Technology, Qingdao, China
[2] National Center for the Molecular Characterization of Genetically Modified Organisms, Joint International Research Laboratory of Metabolic and Developmental Sciences, School of Life Sciences and Biotechnology, Shanghai Jiao Tong University, Shanghai 200240, China
* Correspondence: yylltt@sjtu.edu.cn

Table 1. Primers and TaqMan probes for real-time PCR analysis.

Target	Primer name	Sequences (5′-3′)	Amplicon size (bp)
MON810	Con-MON810-1F	AAACTGCAGCGCGGATCCCAAGGCTTAC ACTCGCTACCAG	892
	Con- MON810-2R	GGAAGATCTGCAGAGGCATCTTCAACGA	
	Q- MON810-1F	CGAAGGACGAAGGACTCTAACG	91
	Q- MON810-2R	GCCACCTTCCTTTTCCACTATCT	
	Q- MON810-P	FAM - CCTTTGCCATTGCCCAGCTATCTGTCACT TT-TAMRA	
zSSIIb	zSSIIb-1F	CGGTGGATGCTAAGGCTGATG	88
	zSSIIb-2R	AAAGGGCCAGGTTCATTATCCTC	
	zSSIIb-P	HEX- TAAGGAGCACTCGCCGCCGCATCTG- TAMRA	
